# Effects of population aging on the mortality burden of related cancers in urban and rural areas of China, 2004–2017: a population-based study

**DOI:** 10.20892/j.issn.2095-3941.2021.0538

**Published:** 2022-03-02

**Authors:** Yali Chen, Cuihong Yang, Ning Li, Zixing Wang, Peng Wu, Jin Du, Jingmei Jiang

**Affiliations:** 1 Department of Epidemiology and Biostatistics, Institute of Basic Medical Sciences, Chinese Academy of Medical Science/School of Basic Medicine, Peking Union Medical College, Beijing 100005, China

**Keywords:** Population aging, cancer, mortality, rural–urban disparity, decomposition

## Abstract

**Objective:**

China is a developing country with urban–rural disparities and accelerating population aging. Therefore, quantifying the effects of population aging on the cancer mortality burden is urgently needed.

**Methods:**

Using data from China’s death surveillance datasets (2004–2017), we decomposed and quantified the effects of population aging and factor variations on cancer mortality rates in urban and rural China during 2004–2017 through a decomposition method. *R* ratios were used to assess the extent of the mortality decreases attributable to factor variations offsetting the increases attributable to population aging for 4 aging-related cancers (lung, colorectal, esophageal, and stomach cancer).

**Results:**

Overall, population aging has led to continued increases in cancer mortality rates in China during 2004–2017 (mortality rates attributable to population aging: 8.63/100,000 for urban men, 4.21/100,000 for urban women, 11.95/100,000 for rural men, and 5.66/100,000 for rural women). The 4 cancers displayed 3 patterns. The mortality rates from lung cancer in rural China and from colorectal cancer nationwide increased because of both population aging and factor variations. Population aging was primarily responsible for the growing mortality due to lung cancer in urban areas. However, for esophageal and stomach cancer, the effect of population aging was not dominant, thus resulting in decreases in mortality rates.

**Conclusions:**

Health resource allocation should prioritize areas or cancers more adversely affected by population aging. The burden of cancer will continue to increase in the future, because of rapid population aging, but can still be offset or even reversed with enhanced cancer control and prevention.

## Introduction

The cancer burden has increased globally, particularly in developing countries. In 2020, the number of global cancer deaths rose to nearly 10.0 million, 30% (3.0 million) of which occurred in China^1^. China’s cancer mortality rate has increased over the past few decades, thus making cancer the leading cause of death in China in 2010^2,3^. In contrast, the cancer mortality rates in developed countries have decreased, with a total decline of 31% from 1991 to 2018 in the United States^4^, and a decrease of approximately 9% over the past decade in the United Kingdom^5^. Despite China’s rising cancer mortality, the age-standardized mortality rate decreased substantially over the period, thus suggesting that population aging is a key driver of the increase in cancer mortality^6^. World Population Prospects 2019 has stated that the proportion of people 65 years of age and older in China has nearly doubled in the past 2 decades, from 6.81% in 2000 to 11.97% in 2020, and is expected to reach 23.7% by 2040^7^. Cancer is largely a disease of old age^8^ and therefore is a major health challenge for China in the 21st century with the aging of the population.

Most studies examining trends in cancer mortality, or making comparisons among countries or regions, have used the age-standardized mortality rate to enable controlling for the effects of population age structure. However, age-standardized mortality rates cannot be used to decompose and quantify the effects of population aging. Several studies have attempted to decompose and quantify the effects of population aging on the disease burden^9–11^. For example, Cheng et al. have disaggregated and quantified the effects of population aging on mortality for 195 countries/territories and 169 causes of death^12^. Li et al. have decomposed and estimated the total deaths attributable to population aging in China. Their results have indicated that the decrease in deaths as a result of changes in other factors that decreased disease mortality partially balanced the increase in deaths due to population aging between 1990 and 2017^13^. However, few studies have quantified the effects of population aging on cancer burden, thereby constraining policymakers’ attempts to address this challenge and improve the healthcare system.

China is a developing country characterized by large rural–urban disparities in factors such as access to healthcare, quality of health services, and cancer survival rates, which are poorer in rural parts of the country^14,15^. In this study, we aimed to decompose and quantify the effects of population aging on the changes in the mortality rates of aging-related cancers in urban and rural areas of China from 2004 to 2017, to provide data-driven evidence for supporting tailored optimization of cancer control and prevention efforts in countries/regions with ongoing and future accelerated population aging.

## Materials and methods

### Data source

Population data and cancer death certification data were obtained from China’s death surveillance datasets (2004–2017)^16^, stratified by sex and age, for urban and rural areas. The datasets are based on the country’s disease surveillance points system, which was established in the 1980s and is considered to provide the most representative information available on national mortality patterns. The coverage ranged from 6% of the total population in 2004 to 24% in 2017. Cancers were classified according to the International Classification of Diseases, Tenth Revision (ICD-10)^17^.

### Selection of aging-related cancers

Deaths due to the 5 cancers with the highest mortality rates in China account for more than two-thirds of all cancer deaths^18^. We therefore selected the 5 cancers with the highest mortality rates separately for men and women, then combined them as major cancers (**Supplementary Table S1**). Six cancers (lung, liver, stomach, esophageal, colorectal, and breast cancer) were identified as major cancers and used in the subsequent selection process.

To confirm that population aging is a major risk factor for cancer and that cancer mortality has not shifted to younger generations^19^, we defined aging-related cancers according to the following criteria: 1) the cancer’s mortality rate in people 65 years and older was more than twice that in other age groups (0–34, 35–49, and 50–64 years); 2) no evidence indicated that the mortality rates in people 65 years of age and older were decreasing and the rates in any of the other 3 age groups were increasing. Liver and breast cancer were excluded because the mortality rates for these 2 cancers in people 65 years and older were less than twice those in the 50–64 age group (**Supplementary Figure S1**). Thus, data on 4 cancers (lung, colorectal, esophageal, and stomach cancer) remained for analysis in this study.

### Statistical analysis

Age-standardized cancer mortality rates for urban and rural areas were calculated by using the 2010 China standard population, obtained from China’s death surveillance datasets. The aging-associated population attributable fraction (*PAF*) was calculated with Levin’s formula^20^:

$$PAF=\frac{p.(RR-1)}{p.(RR-1)+1},$$

where *p* is the proportion of the population 65 years of age and older in the total population, and *RR* is the ratio of mortality rates in the population 65 years of age and older to those in the population 0–64 years of age.

The decomposition method for mortality rates in this study was developed on the basis of the decomposition method for the number of deaths introduced by Cheng et al., because their method offers more justifiable attribution results than other methods^21^. We decomposed the change in mortality rates between 2004 (baseline reference) and each year from 2005 to 2017 into 2 components: mortality rates attributable to population aging and those attributable to factor variations (corresponding to changes in age-specific mortality rates). These factors could be any factors except population aging, including both protective or risk factors, that were associated with changes in mortality rates. We used the following formulas for this process:

$$M_{a}=\sum_{i=1}^{18}(s_{i2}-s_{i1})m_{i1}$$

$$M_{f}=\sum_{i=1}^{18}s_{i1}(m_{i2}-m_{i1})$$

$$I_{af}=\sum_{i=1}^{18}(s_{i2}-s_{i1})(m_{i2}-m_{i1}),$$

where *M_a_* and *M_f_* indicate the main effects of the 2 components, population aging and factor variations; *I_af_* represents the two-way interaction of the 2 components; and *m_ij_* and *s_ij_* denote the age-specific mortality rate and proportion of the population in the *i*th age group (, divided into 18 age groups in 5-year increments from 0–4 years of age to 85+ years of age) of the *j*th year (). The change in mortality rates attributable to population aging and factor variation is as follows:

$$A=M_{a}+\text{½}I_{af}$$

$$F=M_{f}+\text{½}I_{af},$$

where *A* is the mortality rate attributable to population aging, and *F* is the mortality rate attributable to factor variation. We used *R* ratios to assess the comparative effects of population aging *vs.* factor variations on cancer mortality rates. The *R* ratio was calculated as^12^:

$$R\;ratio=\frac{F}{A},$$

where the value of the *R* ratio is (−∞, +∞). *R* < −1 indicates that the increase in the mortality rate attributable to population aging is less than the decrease resulting from variations in other factors; −1 < *R* < 0 indicates that the effects of population aging are larger than the effects of factor variations on decreasing the mortality rate; and R > 0 indicates that population aging and factor variations are both associated with increased mortality rates. Details regarding the decomposition method are provided in the **Supplementary Text**.

**Figure 1:**
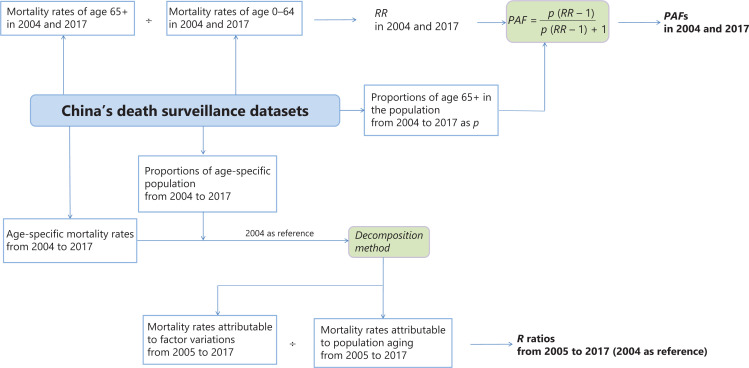
Conceptual framework.

The conceptual framework for statistical analysis is shown in **Figure 1**. All analyses were performed in SAS software, version 9.4 (SAS Institute, Cary, NC, USA).

## Results

### Changes in cancer mortality rates during 2004–2017

Overall, the proportion of the population 65 years of age and older in China increased during 2004–2017, from 8.55% to 11.11% in urban areas, and from 7.53% to 11.61% in rural areas^16^. The mortality rate (per 100,000) for aging-related cancers increased by 1.52 in urban areas and 3.21 in rural areas. The age-standardized rate (per 100,000) decreased by 4.53 in urban areas and 5.68 in rural areas over the same period. The *PAF* associated with aging for these cancers increased from 60.86% to 65.33% in urban areas and 55.93% to 62.84% in rural areas (**Table 1**).

**Table 1:** Aging-related cancer mortality rates in urban and rural China in 2004 and 2017

Area and cancer site	Year	Crude mortality rate (1/10^5^)	Age-standardized mortality rate^†^ (1/10^5^)	Mortality rate of age 65+ (1/10^5^)	*PAF* (%)
Urban					
Aging-related cancer^‡^	2004	21.03	23.00	157.94	60.86
	2017	22.55	18.47	140.39	65.33
Lung	2004	39.80	43.34	301.40	61.44
	2017	48.42	39.77	296.78	64.12
Colorectal	2004	9.83	10.81	74.59	61.57
	2017	13.29	10.82	84.79	67.26
Esophageal	2004	10.98	12.11	84.97	63.00
	2017	11.03	9.00	71.65	68.70
Stomach	2004	23.51	25.75	170.81	58.58
	2017	17.45	14.31	108.33	65.08
Rural					
Aging-related cancer	2004	18.79	23.35	147.84	55.93
	2017	22.00	17.67	127.25	62.84
Lung	2004	25.31	31.39	193.65	54.17
	2017	43.78	35.24	248.59	61.45
Colorectal	2004	6.04	7.44	46.22	54.18
	2017	9.81	7.93	55.48	61.15
Esophageal	2004	17.73	22.09	143.25	57.68
	2017	13.24	10.49	81.37	67.59
Stomach	2004	26.10	32.46	208.23	56.85
	2017	21.18	17.02	123.58	63.51

^†^Age-standardized mortality rates were standardized with the 2010 China standard population. ^‡^Aging-related cancers were defined as all lung, colorectal, esophageal, and stomach cancers. *PAF*, population attributable fraction.

**Table 1** shows the rural–urban disparities in cancer mortality rates. Mortality rates for lung and colorectal cancers were consistently higher in urban than rural areas. However, the mortality rates for esophageal and stomach cancers were consistently lower in urban areas than in rural areas. Cancer mortality rates increased more rapidly in rural than urban areas, particularly for lung cancer (relative increase of 21.66% in urban areas and 72.98% in rural areas). Age-standardized rates decreased more in rural than urban areas, particularly for esophageal cancer (relative decrease of 25.68% in urban areas and 52.51% in rural areas).

**Table 2** shows the age structure and age-specific mortality rates for aging-related cancers in urban and rural areas for both sexes. Compared with that in 2004, the proportion in 2017 was lower for the 2 age groups under 39 years of age and higher for the 5 age groups over 40 years of age for both sexes, and for rural and urban areas. Cancer age-specific mortality rates increased with age.

**Table 2:** Age-specific mortality rates of aging-related cancers^†^ and age structure in urban and rural China in 2004 and 2017

Area and age group (years)	Male				Female			
	2004		2017		2004		2017	
	Mortality rate	Age proportion	Mortality rate	Age proportion	Mortality rate	Age structure	Mortality rate	Age structure
	(1/10^5^)	(%)	(1/10^5^)	(%)	(1/10^5^)	(%)	(1/10^5^)	(%)
Urban								
0–29	0.21	41.81	0.15	37.05	0.18	40.89	0.09	35.69
30–39	3.04	19.26	1.25	15.12	1.81	19.10	1.01	15.05
40–49	12.96	16.34	5.78	19.09	6.39	16.23	3.34	19.11
50–59	41.32	10.85	31.16	13.41	17.34	10.83	11.12	13.36
60–69	109.14	6.94	111.14	8.45	45.01	7.17	37.19	8.60
70–79	254.73	3.81	181.32	5.03	112.18	4.27	74.46	5.74
≥80	346.91	0.99	347.54	1.84	167.39	1.50	189.65	2.44
Rural								
0–29	0.26	48.25	0.13	39.91	0.19	47.12	0.14	37.66
30–39	2.77	18.48	1.89	12.69	1.90	18.63	1.14	12.86
40–49	13.30	13.52	7.47	18.33	5.94	13.54	3.68	18.94
50–59	50.96	9.39	32.88	12.76	20.96	9.28	12.41	12.83
60–69	119.83	6.20	102.80	9.34	50.40	6.18	37.31	9.49
70–79	232.85	3.24	184.07	5.08	104.68	3.80	77.15	5.51
≥80	276.66	0.92	269.65	1.88	153.26	1.45	131.39	2.71

^†^Aging-related cancers were defined as all lung, colorectal, esophageal, and stomach cancers.

The age structure changed more in rural than urban areas during 2004–2017. For example, a greater increase was observed in the proportion of the population 60–69 years of age (1.51% for men and 1.43% for women in urban areas; 3.14% for men and 3.31% for women in rural areas). The proportion of women in the 3 age groups over 60 years of age remained higher than the proportion of men, particularly for those 80 years of age or older in rural areas (from 0.92% in 2004 to 1.88% in 2017 for men; from 1.45% in 2004 to 2.71% in 2017 for women). Age-specific mortality rates for aging-related cancers were consistently lower for women than men in both urban and rural areas.

### Mortality rates attributable to population aging versus factor variations

**Figure 2:**
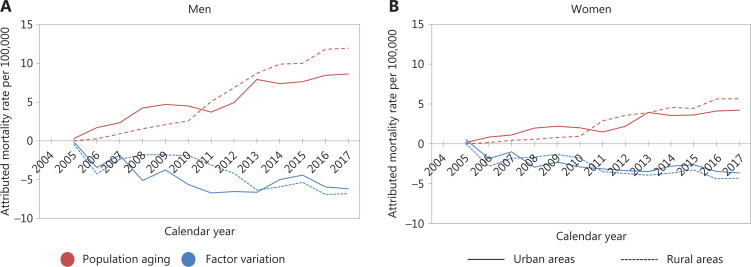
Mortality rates attributable to population aging and factor variations for aging-related cancers^†^ among (A) men and (B) women in urban and rural China from 2005 to 2017 (with 2004 as a reference). ^†^Aging-related cancers were defined as all lung, colorectal, esophageal, and stomach cancers.

As shown in **Figure 2**, population aging resulted in a continued increase in the mortality rates for aging-related cancers over the study period. With 2004 as the baseline reference, we observed a continually increasing mortality rate attributable to population aging (per 100,000) for aging-related cancers during 2004–2017. The increase was more rapid in rural than urban areas, and for men than women, with increases of 0.66 per year for men and 0.32 per year for women in urban areas, and 0.92 per year for men and 0.44 per year for women in rural areas. For aging-related cancers overall, variations in other factors decreased the mortality rate; however, the effect was less than that of population aging on increasing the mortality rate.

For specific cancers, the mortality rates attributable to population aging all increased gradually, but the mortality rates attributable to factor variations varied widely (**Supplementary Figure S2**). The *R* ratios showed 3 patterns. As shown in **Table 3**, the *R* ratios for lung cancer in rural areas were 0.31 for men and 0.28 for women, thus indicating that both population aging and factor variations had negative effects and led to rising mortality. The same situation was observed for colorectal cancer (except in women in urban areas). The *R* ratio for lung cancer in urban men was −0.26. Compared with that in 2004, an increase of 12.88 in the mortality rate (per 100,000) in 2017 was composed of 17.33 from population aging and −4.45 from factor variations. This result indicated that population aging was primarily responsible for the growing mortality burden. The same finding was observed for lung cancer and colorectal cancer in urban women, and esophageal cancer in urban men. The *R* ratios for stomach cancer were −1.97 for men and −2.01 for women in urban areas, and −1.37 for men and −1.63 for women in rural areas, thus indicating that population aging was not the main factor driving the changes in mortality rates. Instead, the rates were offset by variations in other factors, thus resulting in large declines in mortality rates. Similar findings were observed for esophageal cancer (except in men in urban areas).

**Table 3:** Mortality rates attributable to population aging *vs.* factor variations in urban and rural China in 2017 (with 2004 as a reference)

	Male					Female				
Area and cancer site	Crude mortality rate		Attributed mortality rate		*R* ratio	Crude mortality rate		Attributed mortality rate		*R* ratio
	(1/10^5^)		(1/10^5^)			(1/10^5^)		(1/10^5^)		
	2004	2017	Population aging (a)	Factor variation (b)	(c) = (b)/(a)	2004	2017	Population aging (a)	Factor variation (b)	(c) = (b)/(a)
	(reference)					(reference)				
Urban								
Aging-related cancer^†^	27.99	30.44	8.63	−6.18	−0.72	13.87	14.41	4.21	−3.67	−0.87
Lung	53.69	66.57	17.33	−4.45	−0.26	25.49	29.71	8.03	−3.81	−0.47
Colorectal	11.11	15.62	4.14	0.37	0.09	8.51	10.89	2.87	−0.50	−0.17
Esophageal	15.56	16.04	4.78	−4.30	−0.90	6.26	5.86	1.99	−2.39	−1.20
Stomach	31.58	23.53	8.28	−16.33	−1.97	15.19	11.20	3.97	−7.97	−2.01
Rural								
Aging-related cancer	24.74	29.88	11.95	−6.81	−0.57	12.51	13.85	5.66	−4.31	−0.76
Lung	34.09	59.81	19.60	6.13	0.31	16.04	27.19	8.74	2.41	0.28
Colorectal	6.81	11.46	3.76	0.89	0.24	5.22	8.10	2.65	0.22	0.08
Esophageal	23.91	19.37	10.04	−14.58	−1.45	11.20	6.90	4.33	−8.62	−1.99
Stomach	34.17	28.90	14.39	−19.66	−1.37	17.56	13.20	6.90	−11.26	−1.63

^†^Aging-related cancers were defined as all lung, colorectal, esophageal, and stomach cancers.

**Table 3** also shows the rural–urban disparities in mortality rates attributable to factor variations, particularly for lung and esophageal cancers. For lung cancer, factor variations led to a decrease in mortality rates in urban areas (−4.45/100,000 for men, −3.81/100,000 for women) but an increase in rural areas (6.13/100,000 for men, 2.41/100,000 for women). For esophageal cancer, factor variations resulted in a decrease in mortality rates for men in both areas (−4.30/100,000 for urban men, −14.58/100,000 for rural men). The decrease in mortality rates due to factor variation far exceeded the increase due to population aging in rural men (*R* ratio −1.45). However, the effects of variations in other factors were less than that of population aging in urban men (*R* ratio −0.90).

## Discussion

This study reports the first attempt to quantify the effects of population aging on the mortality burden for aging-related cancers in urban and rural areas of China, a country with a rapidly aging society. Population aging resulted in continued growth in the cancer mortality rates during 2004–2017 in both urban and rural areas, and in both sexes. This increase was greater than the decrease in mortality rates attributable to variations in other factors. The 4 investigated cancers were all associated with population aging, but the mortality burdens of these cancers showed different patterns depending on the extent to which factor variations offset the increases attributable to population aging. Lung cancer in rural China and colorectal cancer nationwide were negatively affected by both population aging and factor variations. However, population aging had the dominant influence on lung cancer mortality rates in urban areas. In contrast, population aging was not the main factor influencing esophageal and stomach cancer, for which the increased mortality was offset by a decline in mortality attributable to factor variations.

We used the same selection criteria for aging-related cancers as those used in a previous study focusing on the cancer incidence burden with population aging, and we found that the cancer selection results were consistent. Liver and breast cancers were excluded because their incidence/mortality rates in people 65 years of age and older were not more than twice those in the other age groups^19^. The relative risk (i.e., the ratio of mortality rates in people 65 years of age and older to those in other age groups) must be at least 2 to be statistically significant^22^. We therefore concluded that the population aging effect on liver and breast cancers was not significant. Previous studies have also shown that liver and breast cancers are likely to occur and cause death at relatively young ages^23–26^. In this case, aging-associated *PAF*s would not apply, because 65 years was used as the age cut-off value. Rather than using aging-associated *PAF*s and an age cut-off value of 65 years, we used mortality rates and proportions for each age group to calculate the mortality rate attributable to population aging and factor variations. Attributed mortality rates can be applied to any cancers without considering the significance of the population aging effect; however, the effects of population aging on the cancer mortality burden were of interest only for the related cancers in this study.

Lung cancer, the leading cause of cancer deaths globally, was responsible for 25% of all cancer deaths in 2020^27^. The number of global lung cancer deaths in 2020 was 1,791,644, of which nearly 40.0% (714,699) occurred in China, followed by the United States with 7.7% (138,255) and Japan with 4.6% (82,369)^28^. In the United States, lung cancer remains a concern, and it accounted for approximately 138,225 deaths in 2020, exceeding the number of deaths due to colorectal, liver, and breast cancers combined, despite the substantial decrease in the age-standardized mortality rate of lung cancer after decades of successful tobacco control efforts^4,29^. In Japan, the prevalence of smoking has decreased gradually since 1949 (particularly in men), and the age-standardized mortality rate for lung cancer began to decrease after 1996^30^. In contrast to the decreases in smoking prevalence during 2007–2018 in the United States (a decline of 4%) and Japan (6.4%), the smoking prevalence in China has decreased slowly (2.6%) over the same period^31^. In our study, the age-standardized mortality rate (per 100,000) for lung cancer decreased by 3.57 in urban areas and increased by 3.85 in rural areas during 2004–2017. Specifically, factor variations led to a decrease in the lung cancer mortality rate in urban areas but to an increase in rural areas. This finding may be partially explained by the increase in smoking prevalence during 1983–1996 and subsequent restrictions imposed on smoking in public places, particularly in urban areas^32–34^. Lung cancer control and prevention measures are insufficient, particularly in rural parts of China: the mortality rate of lung cancer still increased even after isolation of the effects of population aging.

With accelerated industrialization and socioeconomic transformation, cancer risk factors in China have changed dramatically over the past 3 decades^35^. Higher intake of fresh vegetables and fruits, improved food preservation practices, and declining levels of chronic *Helicobacter pylori* infection have substantially contributed to the decline in mortality rates from infection- and poverty-associated cancers, including esophageal and stomach cancers^36–38^. Our study indicated that the decrease in mortality rates for esophageal and stomach cancers attributable to factor variations outweighed the increase in mortality rates attributable to population aging. Nevertheless, the mortality burdens of these 2 cancers remain high in China. In 2020, China had the third highest age-standardized stomach cancer mortality rate (15.9/100,000), after Mongolia (24.6/100,000) and Tajikistan (19.7/100,000)^39^, and had the fourth highest standardized esophageal cancer mortality rate (12.7/100,000), after Malawi (16.7/100,000), Mongolia (16.2/100,000), and Bangladesh (13.9/100,000)^40^. The mortality rates for both these cancers were higher in rural areas. Rural–urban disparities, in which rural areas have lower socioeconomic levels, scarcity of medical resources, greater nutritional deficiencies, poorer sanitary conditions, and higher prevalence of *H. pylori* infections, have led to a higher burden of esophageal and stomach cancers in rural parts of the country^15,41–44^. China is undergoing a transition to a westernized lifestyle, with higher consumption of red/processed meat and increased obesity and sedentary lifestyles. This transition has led to an increase in mortality rates for westernized lifestyle-related cancers, including colorectal cancer, which are highly prevalent in most developed countries^3,38,45^. In this study, the colorectal cancer mortality rate showed a worrying trend: both population aging and variations in other factors were responsible for an increase in the mortality rates of colorectal cancer in both rural and urban areas. Like other countries that are undergoing or have undergone a transition from developing to developed economies, China faces daunting challenges in addressing the emerging burden of westernized lifestyle-related cancers and a high pre-transition traditional cancer burden^46,47^.

Cancer screening started later in China than in developed countries. The American Cancer Society has issued cancer screening guidelines since 1980^48^. The Europe Against Cancer program, which focuses on cancer prevention, screening, and education, was launched in 1985^49^. France began implementing a colorectal cancer screening program in 2003, which might have contributed to the decline in the colorectal cancer mortality rate in France, from 35.81/100,000 in 2003 to 34.96/100,000 in 2014^49,50^. In China, the government-supported Cancer Screening Program in Rural Areas, which focuses on upper gastrointestinal tract cancers, has offered screening in some high-risk rural areas since 2005. This initiative was followed by the Cancer Screening Programs in Urban Areas, which have aimed to screen for upper gastrointestinal, liver, colorectal, lung, and breast cancers among high-risk populations in urban China since 2012^51^. An earlier start to cancer screening in rural areas might partially explain the greater decrease in mortality rates for esophageal and stomach cancers in rural than urban parts of China in this study. A population-based multicenter cohort study has estimated the effectiveness of an endoscopic screening program, and has also reported a significant decrease in the incidence and mortality of all types of upper gastrointestinal cancers in rural areas during 2005–2015^52^. Because China is a developing country with major rural–urban disparities and limited health resources, optimal health resource allocation is more cost-effective in long-term cancer control than having full coverage across the entire population.

Our findings may help policymakers identify cancers and areas with a relatively severe mortality burden, such as those negatively affected by both population aging and variations in other factors, to tailor local healthcare provision to population needs. The *R* ratio is an important indicator for quantifying the proportion of changes in cancer mortality attributable to population aging; more importantly it can be used to assess the contributions of underlying risk factors that change over time. These factors are precisely those that can be controlled through effective measures. Our study therefore provides important evidence that, although the cancers investigated in this study are all associated with population aging, the patterns of the comparative effects of population aging and factor variations appear to differ across cancers and areas, according to the *R* ratios. This study therefore makes a significant contribution; however, it has several limitations. First, our results were dependent on the quality of China’s death surveillance datasets. The datasets covered only 6% of the total population before 2010, thus potentially resulting in poorer data quality for the 2004–2010 period than the 2010–2017 period, and influencing the cancer mortality rates. Second, the age structure was calculated from the datasets based on the population in the disease surveillance point system. The population count data by sex and age (in each 5-year age group, from 0–4 to 85+ years of age), stratified according to urban and rural areas of China, were not available. We therefore were unable to compare the effects of population aging and factor variations on the cancer burden according to the number of attributed deaths. Finally, most cancers are associated with population aging in some way, but we focused on only 4 aging-related cancers with relatively high mortality rates in China. Although not of interest in this study, population aging also influences cancers with relatively low mortality rates.

## Conclusions

Population aging has led to an increase in cancer mortality in China, and this burden will continue to increase in the future because of the rapidly increasing proportion of older people in the country’s population. Wide variations exist in the comparative effects between population aging and changes in other factors across different cancers and areas. We therefore recommend that health resources be allocated to prioritize cancers and areas where the effects of population aging outweigh the mortality reduction due to factor variations, or even factor variations associated with the increasing mortality rate. The burden of population aging in China is inevitable and growing, but its effects on cancer can still be offset or even reversed with enhanced cancer control and prevention measures.
