# Supplementary material for: Effects of population aging on the mortality burden of related cancers in urban and rural areas of China, 2004–2017: a population-based study
Source: Cancer Biol Med. 2022 Mar 2;19(5):696–706. doi: 10.20892/j.issn.2095-3941.2021.0538 (PMC9196052; doi:10.20892/j.issn.2095-3941.2021.0538)
Supplement: Supplementary file 1 [file cbm-19-696-s001.pdf]

Supplementary materials

Supplementary text: Decomposition method

This method was used to decompose the differences in mortality rates over calendar years into the factor variations and age structure, generalized from a method for decomposing the differences in number of deaths into mortality changes (equivalent to factor variations in this study), age structure, and population size (two factors for rate differences and 3 factors for count differences)[1].

With the difference in mortality rates for lung cancer between 2004 and 2017 in men from urban areas of China used as an example, the calculation of mortality attributable to the 2 factors was as follows. Age was divided into 5-year increments, from age 0–4 years to ≥85 years. Let m_{ij} and s_{ij} denote the age-specific mortality rate and proportion of the population for the *i*th age group of the *j*th year ($i = 1, 2, \ldots, 18$; $j = 1, 2$). Let M_1 and M_2 represent mortality rates for the years 2004 and 2017 (**Supplementary Table S2**).

We used M_a and M_f to represent the main effects of changes in age structure and age-specific mortality rates (factor variations) and I_{af} to represent their two-way interactions. These terms were calculated as follows, by using 2004 as the reference year:

$$M_a = \sum_{i=1}^{18} \left(s_{i2} - s_{i1}\right) m_{i1}$$

$$M_f = \sum_{i=1}^{18} s_{i1} \left(m_{i2} - m_{i1}\right)$$

Table S1 Mortality rates of the 5 cancers with the highest mortality rates in China in 2017

Rank	Total			Male			Female		
	Cancer site	ICD-10[†]	Mortality rate ($1/10^5$)	Cancer site	ICD-10	Mortality rate ($1/10^5$)	Cancer site	ICD-10	Mortality rate ($1/10^5$)
1	Lung	C33-34	45.35	Lung	C33-34	62.09	Lung	C33-34	28.05
2	Liver	C22	25.17	Liver	C22	36.60	Liver	C22	13.36
3	Stomach	C16	19.92	Stomach	C16	27.09	Stomach	C16	12.52
4	Esophageal	C15	12.49	Esophageal	C15	18.24	Colorectal	C18-21	9.04
5	Colorectal	C18-21	10.99	Colorectal	C18-21	12.87	Breast	C50	7.81

[†]ICD-10, International Classification of Diseases, Tenth Revision.

Table S2 Mathematical symbols in the decomposition formula

Age group (years)	2004 (j = 1)		2017 (j = 2)	
	Mortality rate	Age proportion	Mortality rate	Age proportion
0–4	m_{11}	s_{11}	m_{12}	s_{12}
5–9	m_{21}	s_{21}	m_{22}	s_{22}
10–14	m_{31}	s_{31}	m_{32}	s_{32}
⋮	⋮	⋮	⋮	⋮
80–84	m_{171}	s_{171}	m_{172}	s_{172}
≥85	m_{181}	s_{181}	m_{182}	s_{182}
Total	M_1	$S_1 = 1$	M_2	$S_2 = 1$

$$I_{af} = \sum_{i=1}^{18} \left(s_{i2} - s_{i1}\right)\left(m_{i2} - m_{i1}\right)$$

With 2017 as the reference year, the formulas were calculated as follows:

$$M'_{a} = \sum_{i=1}^{18} \left(s_{i1} - s_{i2}\right) m_{i2}$$

$$M'_{f} = \sum_{i=1}^{18} s_{i2} \left(m_{i1} - m_{i2}\right)$$

$$I'_{af} = \sum_{i=1}^{18} \left(s_{i1} - s_{i2}\right)\left(m_{i1} - m_{i2}\right)$$

The contribution of each factor includes its main effect and partial interactions with other factors.

Suppose that a% of the two-way interaction between age structure and factor variation is allocated to the first factor. Then $(100 - a)\%$ of the two-way interaction is allocated to the second factor.

With A (A') and F (F') representing the mortality rates attributable to age structure and factor variation defined by the method, and 2004 (2017) as the reference year, the contributions of the 2 factors can be calculated as follows:

$$A = M_{a} + a\% I_{af}$$

$$F = M_{f} + \left(100 - a\right)\% I_{af}$$

$$A' = M'_{a} + a\% I'_{af}$$

$$F' = M'_{f} + \left(100 - a\right)\% I'_{af}$$

Table S3 Age-specific mortality rates for lung cancer and age structure among men from urban China in 2004 and 2017

Age group (years)	2004		2017	
	Mortality rate (1/10^5)	Age proportion (%)	Mortality rate (1/10^5)	Age proportion (%)
0–4	0.00	4.61	0.00	4.68
5–9	0.00	5.61	0.00	4.95
10–14	0.00	7.19	0.10	4.33
15–19	0.21	7.81	0.11	6.05
20–24	0.42	7.81	0.16	9.57
25–29	1.13	8.77	1.04	7.47
30–34	4.22	9.76	1.79	6.86
35–39	8.51	9.50	2.92	8.25
40–44	18.36	8.26	7.97	8.75
45–49	34.21	8.07	15.57	10.35
50–54	62.57	6.32	63.70	6.27
55–59	93.39	4.53	76.96	7.14
60–64	152.37	3.76	208.75	4.93
65–69	281.98	3.18	307.80	3.52
70–74	447.83	2.38	357.02	2.79
75–79	574.79	1.43	445.10	2.24
80–84	649.07	0.66	641.55	1.25
≥85	608.32	0.32	885.37	0.59
Total	53.69	100.00	66.57	100.00

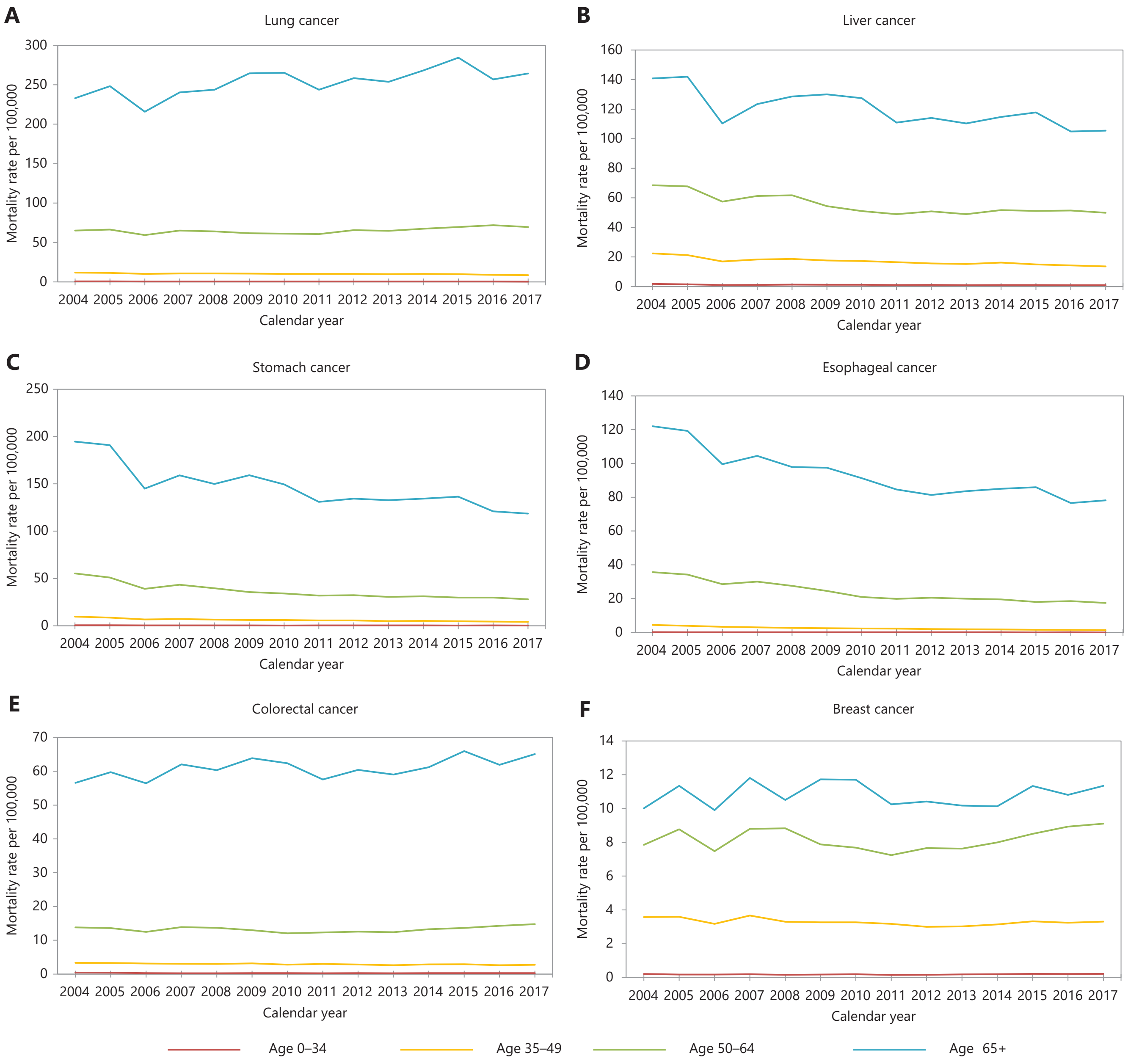

Figure S1 Mortality rates across age groups for (A) lung cancer, (B) liver cancer, (C) stomach cancer, (D) esophageal cancer, (E) colorectal cancer, and (F) breast cancer in China in 2017.

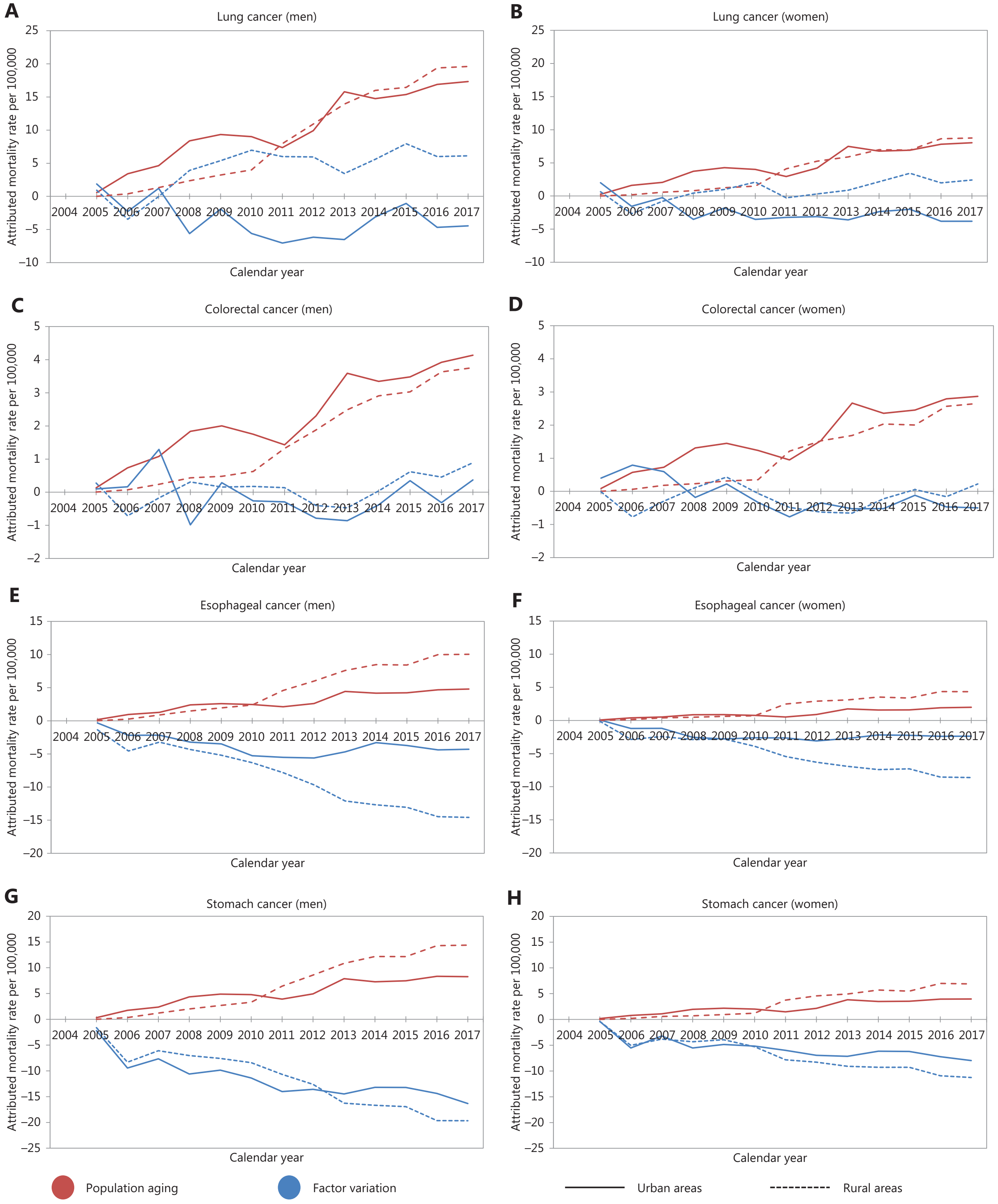

Figure S2 Mortality rates attributable to population aging and factor variations for (A) lung cancer, (B) colorectal cancer, (C) esophageal cancer, and (D) stomach cancer among men, and (E) lung cancer, (F) colorectal cancer, (G) esophageal cancer, and (H) stomach cancer among women, in urban and rural China from 2005 to 2017 (with 2004 as a reference).

The absolute values of the decomposition results should remain unchanged when the reference year changes, thus yielding 2 equations:

$$A \equiv -A'$$
$$F \equiv -F'$$

Through formula derivation, we can generate 2 simplified equations:

$$\sum_{i=1}^{18}\left(s_{i2}-s_{i1}\right)\left[\left(m_{i1}-m_{i2}\right)+2\left(m_{i2}-m_{i1}\right)a\%\right]\equiv 0$$

$$\sum_{i=1}^{18}\left(m_{i2}-m_{i1}\right)\left[\left(s_{i1}-s_{i2}\right)+2\left(s_{i2}-s_{i1}\right)\left(100-a\right)\%\right]\equiv 0$$

These 2 equations cannot be true all the time unless a equals 50.

The contributions of the 2 factors can be calculated as follows:

$$A = M_a + ½I_{af}$$

$$F = M_f + ½I_{af}$$

Thus, A represents the effect of changes in age structure. Because of the relatively higher proportion of older age groups in our study, the effect of age structure represents that of population aging (**Supplementary Table S3**).

An example

$$M_a = \sum_{i=1}^{18}\left(s_{i2}-s_{i1}\right)m_{i1}$$
$$=\left(4.68\%-4.61\%\right)\times 0.00+\cdots+\left(0.59\%-0.32\%\right)$$
$$\times 608.32 = 17.71$$

$$I_{af} = \sum_{i=1}^{18}\left(s_{i2}-s_{i1}\right)\left(m_{i2}-m_{i1}\right)$$
$$=\left(4.68\%-4.61\%\right)\times\left(0.00-0.00\right)+\cdots$$
$$+\left(0.59\%-0.32\%\right)\times\left(885.37-608.32\right)=-0.76$$

$$A = M_a + ½I_{af} = 17.71 - ½\times 0.76 = 17.33$$

The mortality rate attributable to factor variation can be calculated similarly by using the formulas above.

Reference

1. Cheng XJ, Tan LH, Gao YY, Yang Y, Schwebel DC, Hu GQ. A new method to attribute differences in total deaths between groups to population size, age structure and age-specific mortality rate. PLoS One. 2019; 14: e0216613.
